# Prediabetes and diabetes in relation to risk of gastric adenocarcinoma

**DOI:** 10.1038/s41416-019-0470-1

**Published:** 2019-05-07

**Authors:** Jiaojiao Zheng, Martin Rutegård, Giola Santoni, Bengt Wallner, Ingegerd Johansson, Malin Sund, Shao-Hua Xie, Jesper Lagergren

**Affiliations:** 1 Upper Gastrointestinal Surgery, Department of Molecular Medicine and Surgery, Karolinska Institutet, Karolinska University Hospital, Stockholm, Sweden; 2 Faculty of Medicine, Surgery, Department of Surgical and Perioperative Sciences, Umeå University, Umeå, Sweden; 3 Wallenberg Centre for Molecular Medicine, Umeå University, Umeå, Sweden; 4 Faculty of Medicine, Department of Dentistry, School of Dentistry, Cariology, Umeå University, Umeå, Sweden; 5 School of Cancer and Pharmaceutical Sciences, King’s College London, London, United Kingdom

**Keywords:** Risk factors, Epidemiology, Gastric cancer, Diabetes, Risk factors

## Abstract

**Background:**

Whether prediabetes or diabetes increases the risk of gastric adenocarcinoma is not clear.

**Methods:**

This cohort study included 111,198 participants in the Northern Swedish Health and Disease Study. The participants were followed up from November 1985 to April 2017. The exposure to prediabetes or diabetes was assessed by oral glucose tolerance tests and self-reports. The incidence of the outcome gastric adenocarcinoma was identified from the Swedish Cancer Registry. Multivariable Cox regressions were used to analyse the associations between prediabetes or diabetes and the risk of gastric adenocarcinoma, providing hazard ratios (HR) with 95% confidence intervals (CI), with adjustment for sex, age, calendar year, body mass index, tobacco smoking and education level.

**Results:**

Compared with normoglycaemic participants, the risk of gastric adenocarcinoma was not increased among participants with prediabetes (HR 1.07, 95% CI 0.79–1.44), diabetes (HR 0.77, 95% CI 0.46–1.29) or any of these exposures (HR 0.96, 95% CI 0.73–1.27). No associations were identified between prediabetes or diabetes and the risk of gastric adenocarcinoma in stratified analyses or in analyses separating cardia and non-cardia gastric adenocarcinoma.

**Conclusions:**

This study does not support the hypothesis that prediabetes or diabetes increases the risk of gastric adenocarcinoma.

## Background

Gastric cancer (adenocarcinoma in 98% of the cases) is the sixth most common cancer worldwide.^1^ The overall 5-year survival is below 30%.^2^ The anatomical subtypes non-cardia and cardia gastric adenocarcinoma have different aetiology. *Helicobacter pylori* (*H. pylori*) infection is the strongest risk factor for gastric non-cardia adenocarcinoma, while gastro-oesophageal reflux and obesity increase the risk of gastric cardia adenocarcinoma.^3^ Diabetes mellitus, one of the most prevalent diseases worldwide, has recently been found to increase the risk of some cancer types, i.e., cancer of the liver, pancreas and endometrium.^4^ These associations may be mediated by hyperglycaemia, hyperinsulinemia and insulin like growth factor.^4^ Prediabetes, an often unrecognised condition characterised by impaired glucose tolerance, hyperglycaemia, and insulin resistance, may also increase the overall cancer risk, and again specifically the risk of cancer of the liver, pancreas and endometrium.^5^ Some studies have found a higher prevalence of *H. pylori* infection and gastric mucosal atrophy among diabetes patients, implying a potential role of diabetes also in the aetiology of gastric adenocarcinoma.^6,7^ However, epidemiological studies on this topic are limited and the findings have been inconsistent.^8–12^ Major concerns in the existing literature on this topic are confounding by shared risk factors for prediabetes or diabetes and gastric adenocarcinoma, i.e., obesity, smoking and dietary factors,^13^ as well as reverse causality, which can best be avoided by prospective studies with long-term follow-up.^14^

The present study aimed to clarify whether prediabetes and diabetes increase the risk of gastric adenocarcinoma by analysing a cohort with long and complete follow-up and valid information on prediabetes and diabetes, gastric adenocarcinoma and potential confounders.

## Methods

### Cohort

The Northern Sweden Health and Disease Study is a cohort constructed from three sub-cohorts: the Västerbotten Intervention Program (VIP) cohort, the Monitoring Trends and Determinants in Cardiovascular Disease (MONICA) cohort, and the mammary screening cohort, all from the Northern part of Sweden. The sub-cohorts VIP and MONICA were used for the present study because of the availability of data on prediabetes, diabetes and risk factors for gastric adenocarcinoma. These two sub-cohorts have been described in detail elsewhere.^15,16^ In brief, the VIP cohort is an ongoing community intervention programme for diabetes and cardiovascular diseases launched from 1985 in the county of Västerbotten. Each year, all county residents who turn 40, 50, or 60 years of age are invited to their primary healthcare centre, where a detailed physical examination is conducted, blood samples are collected and questionnaires assessing socioeconomic status, mental and physical health, lifestyle factors and dietary habits are completed. In September 2017, 105,700 individuals were enroled into the VIP cohort.

The MONICA cohort is part of a multinational population survey evaluating risk factors for cardiovascular events, coordinated by the World Health Organization (WHO). Inhabitants from the counties of Norrbotten and Västerbotten were invited to attend the survey. Those who agreed to participate were asked to take a physical examination, give blood samples and complete a questionnaire assessing risk factors for cardiovascular diseases. Thus far six surveys have been conducted. In March 2015, 11,800 individuals were enroled in the MONICA cohort.

### Design

This was a cohort study including participants enroled in the VIP or MONICA cohorts between 2 November 1985 and 18 November 2016. Exposure to prediabetes or diabetes and data on potential confounders were assessed as part of the data collection of all cohort participants. The outcome gastric adenocarcinoma was assessed through linkage to the Swedish Cancer Registry, which is 98% complete for the recording of gastric cancer.^17^ The follow-up started from the date of entry into the cohort (date of the first assessment) and ended at the date of gastric adenocarcinoma diagnosis, death, or the end of study period (April 27th of 2017), whichever occurred first. Death was identified from the Swedish Cause of Death Registry, which has 100% completeness for date of death.^18^ Linkage of information for each cohort participant from health data registers was enabled by the unique personal identity numbers of all Swedish residents.

### Exposures

The exposures to prediabetes or diabetes were assessed at entry into the cohort. Prediabetes was measured by an oral glucose tolerance test (blood glucose measured at fasting and 2-h later after a 75 g oral glucose load), which was included among the physical examinations. Diabetes was assessed by the oral glucose tolerance test or self-reported in the questionnaire (participants with known diabetes were exempt from the tolerance test). The WHO standards were used to define prediabetes and diabetes based on the results of the oral glucose tolerance test.^19^ Participants were considered normoglycaemic if their fasting blood glucose level was <6.1 mmol/L and 2-h post-load glucose level was <7.8 mmol/L, and if they did not report diabetes in the questionnaire. In the VIP cohort, a Reflotron bench-top analyser (Roche Diagnostics) was used to test the plasma glucose levels before the year 2004 and a Hemocue bench-top analyser (Quest Diagnostics) was used from 2004 onwards.^16^ In the MONICA cohort, the plasma glucose levels were tested by a hexokinase method (Boehringer Mannheim Automated Analysis for BM/Hitachi System 717).^15^ For individuals included in the VIP cohort, health counselling was provided after the physical examination, and individuals with blood tests indicating diabetes were referred to their general practitioner. For individuals included in the MONICA cohort, those with blood tests suggestive of diabetes were informed on site and encouraged to seek medical attention.

### Outcome

The study outcome was newly diagnosed gastric adenocarcinoma during the follow-up according to the Swedish Cancer Registry. Gastric adenocarcinoma was defined by the anatomical site code 151 in the 7th edition of the International Classification of Diseases (ICD-7), combined with the histology code 096 or 196 in the WHO/HS/CANC/24.1 classification in the Cancer Registry. The sub-sites gastric non-cardia adenocarcinoma (ICD-7 site codes 151, excluding 151.1) and gastric cardia adenocarcinoma (ICD-7 site code 151.1) were also identified from the Cancer Registry.

### Covariates

Twelve covariates were considered as potential confounders because they were risk factors for gastric adenocarcinoma: (1) sex, (2) age (continuous), (3) calendar year of inclusion (1986–1994, 1995–2003, or 2004–2017), (4) body mass index (BMI) (categorised into three equal-sized groups, i.e., tertiles), (5) tobacco smoking status (never smoker, ex-smoker, or current smoker), (6) education level (compulsory school or less, upper secondary school, or higher education or postgraduate level), (7) marital status (married or cohabitating, or living alone), (8) daily alcohol consumption (tertiles), (9) physical activity level defined by the Cambridge physical activity index (active or inactive),^20^ (10) daily intake of fresh fruit and vegetables (tertiles), (11) daily sodium intake (tertiles), and (12) daily energy intake (tertiles). Except for BMI, which was calculated according to the height and weight measured at the first physical examination, data on all other covariates were collected from the questionnaire answered at baseline (cohort entry).

### Statistical analysis

Cox proportional hazard models were used to calculate hazard ratios (HR) with 95% confidence intervals (CI). Age was used as time scale in all analyses and all other covariates were considered for inclusion in a multivariable model. With a stepwise backward selection, covariates that failed to lead to ≥10% change in HR estimate were excluded from the model. After the exclusions, the final model included five covariates: (1) sex, (2) calendar year, (3) BMI, (4) tobacco smoking status and (5) education level. Missing data on covariates were treated as a separate category. Three exposed groups were analysed: prediabetes alone, diabetes alone, and any of the exposures prediabetes or diabetes. For participants who took part in the surveys more than once, their exposure status was allowed to change (time-varyingly). Exposure to prediabetes or diabetes was mutually exclusive. Thus, participants with previous prediabetes were censored from the prediabetes group at the date of a diabetes diagnosis. When analysing prediabetes alone, participants with prediabetes could reverse to normoglycaemic status during the follow-up. In the analyses of the diabetes group and the prediabetes or diabetes combined group, the exposure was regarded as irreversible. The normoglycaemic group was the reference group in all analyses.

The following sub-analyses were conducted: (1) A sensitivity analysis was performed after excluding gastric adenocarcinoma cases diagnosed within the first year of follow-up in order to evaluate possible reverse causality, i.e., earlier detection of gastric adenocarcinoma because of the presence of prediabetes or diabetes. (2) Stratified analyses by sex, age (above or below the median), BMI (above or below the median), and tobacco smoking habits were conducted in the prediabetes or diabetes combined group in order to test for interactions. (3) Non-cardia and cardia gastric adenocarcinoma were analysed separately in the prediabetes group and the prediabetes or diabetes combined group. (4) Continuous fasting glucose levels, as well as 2-h post-load glucose levels were tested for linear trends in relation to the risk of gastric adenocarcinoma.

The proportionality assumption of the hazards in the Cox regression analyses was tested by Schoenfeld residuals and the assumption was met for all analyses. An experienced biostatistician was responsible for the data management and statistical analyses, which followed a pre-defined study protocol. All data management and statistical analyses were conducted using the statistical software Stata (Release 15, StataCorp, College Station, Texas, USA).

## Results

### Participants

The final study cohort included 111,198 participants with median age at cohort entry being 49.8 (interquartile range 40.0–50.6 years). These participants were followed up for a median of 12.2 years (interquartile range 7.5–19.0 years), comprising 1,579,657 person-years of follow-up. At entry, 23,900 (21.5%) participants had prediabetes, 5958 (5.4%) had diabetes, and 81,340 (73.1%) were normoglycaemic. Compared with the normoglycaemic group, both the prediabetes and diabetes groups were older, had higher BMI and lower education level, but included fewer current smokers and heavy drinkers (Table 1). These differences were more pronounced for the diabetes group than the prediabetes group.

**Table 1:** Baseline characteristics of the study participants (*n* = 111,198)

	Normoglycaemic	Prediabetes	Diabetes
Number (%)	81,340 (73.1)	23,900 (21.5)	5958 (5.4)
Sex			
Male	40,481 (49.8)	11,023 (46.1)	3363 (56.5)
Female	40,859 (50.2)	12,877 (53.9)	2595 (43.5)
Age (years)			
Mean ± standard deviation	45.7 ± 8.9	50.0 ± 8.6	53.8 ± 8.8
<40	20,462 (25.1)	3221 (13.5)	473 (7.9)
40–49	32,465 (39.9)	7771 (32.5)	1290 (21.7)
50–59	20,577 (25.3)	8517 (35.6)	2408 (40.4)
60–69	7706 (9.5)	4311 (18.0)	1676 (28.1)
≥70	130 (0.2)	80 (0.3)	111 (1.9)
Year of entry into the cohort			
1986–1996	26,865 (33.0)	6483 (27.1)	1785 (30.0)
1997–2005	27,261 (33.5)	10,111 (42.3)	2169 (36.4)
2006–2017	27,214 (33.5)	7306 (30.6)	2004 (33.6)
Body mass index			
Mean ± standard deviation	25.5 ± 4.0	27.1 ± 4.6	29.4 ± 5.5
≤24	31,747 (39.0)	6025 (25.2)	866 (14.5)
24–27	25,233 (31.0)	6857 (28.7)	1212 (20.3)
≥27	24,164 (29.7)	10,947 (45.8)	3852 (64.7)
Missing	196 (0.2)	71 (0.3)	28 (0.5)
Education level			
Compulsory school or less	14,277 (17.5)	6159 (25.8)	2060 (34.6)
Upper secondary school	41,298 (50.8)	11,575 (48.4)	2729 (45.8)
Higher education/postgraduate	25,129 (30.9)	5917 (24.8)	1043 (17.5)
Missing	636 (0.8)	249 (1.0)	126 (2.1)
Tobacco smoking status			
Never smoker	15,853 (19.5)	4830 (20.2)	1303 (21.9)
Ex-smoker	23,027 (28.3)	7440 (31.1)	2036 (34.2)
Current smoker	41,435 (50.9)	11,263 (47.1)	2475 (41.5)
Missing	1025 (1.3)	367 (1.5)	144 (2.4)
Marital status			
Married or cohabitant	15,524 (19.1)	5204 (21.8)	1586 (26.6)
Living alone	65,233 (80.2)	18,461 (77.2)	4288 (72.0)
Missing	583 (0.7)	235 (1.0)	84 (1.4)
Daily alcohol consumption (milligrams)			
≤9	18,759(23.1)	6695 (28.0)	2041 (34.3)
9–163	30,480 (37.5)	8743 (36.6)	2037 (34.2)
≥163	30,642 (37.7)	8242 (34.5)	1689 (28.3)
Missing	1459 (1.8)	220 (0.9)	191 (3.2)
Physical activity level			
Inactive	35,639 (43.8)	12,017(50.3)	2876 (48.3)
Active	41,381 (50.9)	11,002 (46.0)	2257 (37.9)
Missing	4320 (5.3)	881 (3.7)	825 (13.8)
Daily intake of fruit and vegetables (grams)			
≤1.8	30,979 (33.7)	8430 (33.1)	1895 (31.6)
1.8–3.2	30,194 (32.9)	8210 (32.2)	1812 (30.3)
≥3.2	28,898 (31.4)	8577 (33.7)	2087 (34.9)
Missing	1808 (2.0)	1428 (1.0)	194 (3.2)
Daily sodium intake (milligrams)			
≤1643	27,137 (29.5)	8978 (35.2)	1926 (32.2)
1643–2266	28 870 (31.4)	8082 (31.7)	1739 (29.0)
≥2266	30 377 (33.1)	7195 (28.2)	1719(28.7)
Missing	5 488 (6.0)	1223 (4.8)	604 (10.1)
Daily total energy intake (kilocalories)			
≤1411	27,251 (29.6)	9009 (35.4)	2087(34.9)
1411–1908	29,299 (31.9)	8010 (31.4)	1730 (28.9)
≥1908	30,652 (33.4)	7349 (28.8)	1626 (27.1)
Missing	4670 (5.1)	1110 (4.4)	545 (9.1)
Follow-up (years)			
Median (interquartile range)	13.4 (6.8–20.0)	14.3 (8.2–19.3)	12.9 (7.4–18.5)

### Risk of gastric adenocarcinoma

During the follow-up of all participants, 219 participants (0.2%) developed gastric adenocarcinoma. The risk of gastric adenocarcinoma was not increased in the participants with any of the exposures prediabetes or diabetes compared with the normoglycaemic participants (crude HR 0.97, 95% CI 0.74–1.28; adjusted HR 0.96, 95% CI 0.73–1.27). Exclusion of the 7 gastric adenocarcinoma cases that occurred during the first year of follow-up did not change this risk estimate (crude HR 0.97, 95% CI 0.74–1.29; adjusted HR 0.96, 95% CI 0.72–1.27).

In separate analyses, neither the prediabetes group (crude HR 1.01, 95% CI 0.81–1.45; adjusted HR 1.07, 95% CI 0.79–1.44), nor the diabetes group (crude HR 0.83, 95% CI 0.50–1.38; adjusted HR 0.77, 95% CI 0.46–1.29), had any increased risk of gastric adenocarcinoma compared with the normoglycaemic group. No associations were identified in the analyses stratified by sex, age, BMI, or tobacco smoking habits (Table 2), or in the analyses separating cardia and non-cardia gastric adenocarcinoma (Table 3). Finally, there was no linear association between the plasma values of fasting glucose (adjusted HR 1.02, 95% CI 0.91–1.14), or post-load glucose (adjusted HR 0.98, 95% CI 0.90–1.05) and the risk of gastric adenocarcinoma.

**Table 2:** Risk of gastric adenocarcinoma in participants exposed to prediabetes or diabetes compared with normoglycaemic participants, stratified by covariates, expressed as hazard ratio (HR) and 95% confidence interval (CI)

Stratified variable	Gastric adenocarcinoma	Crude model^a^	Multivariable model^b^
	Number	HR (95% CI)	HR (95% CI)
Sex			
Male			
Normoglycemic	82	Reference	Reference
Prediabetes or diabetes	45	0.81 (0.56–1.17)	0.80 (0.55–1.16)
Female			
Normoglycemic	50	Reference	Reference
Prediabetes or diabetes	42	1.24 (0.82–1.88)	1.20 (0.79–1.84)
Age at entry			
≤49.5			
Normoglycemic	25	Reference	Reference
Prediabetes or diabetes	10	0.83 (0.39–1.77)	0.90 (0.42–1.93)
>49.5			
Normoglycemic	107	Reference	Reference
Prediabetes or diabetes	77	1.00 (0.75–1.35)	0.98 (0.73–1.33)
Body mass index			
≤25.4			
Normoglycemic	68	Reference	Reference
Prediabetes or diabetes	34	1.05 (0.70–1.60)	1.05 (0.69–1.59)
>25.4			
Normoglycemic	64	Reference	Reference
Prediabetes or diabetes	53	0.90 (0.62–1.30)	0.90 (0.63–1.31)
Tobacco smoking status			
Current smoker			
Normoglycemic	39	Reference	Reference
Prediabetes or diabetes	33	1.22 (0.77–1.95)	1.21 (0.75–1.94)
Ex-smoker			
Normoglycemic	41	Reference	Reference
Prediabetes or diabetes	24	0.89 (0.54–1.48)	0.81 (0.48–1.36)
Non-smoker			
Normoglycemic	50	Reference	Reference
Prediabetes or diabetes	26	0.78 (0.48–1.26)	0.81 (0.50–1.32)

^a^Age was used as the time scale

^b^Adjusted for sex, calendar year, BMI, tobacco smoking status and education level at baseline; age was used as the time scale

**Table 3:** Risk of gastric cardia and non-cardia adenocarcinoma in participants exposed to prediabetes or diabetes and in those exposed to prediabetes compared with normoglycaemic participants, expressed as hazard ratio (HR) and 95% confidence interval (CI)

Exposure and outcome	Gastric adenocarcinoma	Crude model^a^	Multivariable model^b^
	Number	HR (95% CI)	HR (95% CI)
Cardia adenocarcinoma			
Normoglycaemic	43	Reference	Reference
Prediabetes or diabetes	27	0.96 (0.59–1.56)	0.94 (0.57–1.54)
Non-cardia adenocarcinoma			
Normoglycaemic	89	Reference	Reference
Prediabetes or diabetes	60	0.98 (0.70–1.36)	0.97 (0.69–1.35)
Cardia adenocarcinoma			
Normoglycaemic	43	Reference	Reference
Prediabetes	25	1.28 (0.78–2.11)	1.28 (0.78–2.12)
Non-cardia adenocarcinoma			
Normoglycaemic	92	Reference	Reference
Prediabetes	42	0.97 (0.67–1.40)	0.97 (0.67–1.40)

^a^Age was used as the time scale

^b^Adjusted for sex, calendar year, BMI, tobacco smoking status and education level at baseline; age was used as the time scale

## Discussion

This study found no support for the hypothesis that prediabetes or diabetes increases the risk of gastric adenocarcinoma. The lack of association remained in sub-analyses stratified by covariates, excluding the gastric adenocarcinoma cases occurring within the first year of follow-up, separating non-cardia and cardia gastric adenocarcinomas, and in linear analyses of blood glucose levels.

Strengths of this study include the prospective design, comprehensive information on exposures, confounders and the outcome, as well as the long and complete follow-up. The objectively measured glucose levels enabled the analyses of prediabetes in relation to the risk of gastric adenocarcinoma. The study also has weaknesses. Due to the low incidence of gastric adenocarcinoma in Sweden,^21^ the statistical power was limited, and weak associations might have been missed. Moreover, despite the fact that twelve potential confounders were considered in this study, residual confounding cannot be excluded because data on some other potentially relevant factors were not available, e.g., *H. pylori* infection and medication. *H. pylori* infection is the major known risk factors for gastric cancer, but no established associations has been found between prediabetes or diabetes and *H. pylori* infection.^22^ Thus, H. pylori cannot influence the association between diabetes and risk of gastric cancer. Accordingly, a Japanese cohort study showed that adjustment of *H. pylori* did not change the associations between hyperglycaemia and the risk of gastric cancer.^12^ Metformin is a first-line medication for treating diabetes and has been shown to decrease the risk of several cancers, but existing evidence regarding metformin use and gastric adenocarcinoma remains inconclusive.^23,24^ Moreover, the majority of the ‘exposed’ participants in this study were those with prediabetes rather than diabetic patients, and thus, were less likely to have used metformin. Taken together, lack of data on these factors should not be a threat to the validity of the findings.

Studies examining the association between diabetes and risk of gastric adenocarcinoma have provided inconsistent results.^9–12^ Differences in study populations, exposure measurements, lengths of follow-up, and confounders adjusted for, might explain the varying findings. Particularly the exposure assessments varied considerably across studies, including self-reported diagnosis,^25^ diagnosis identified from medical records, healthcare registries or health insurance claims,^26–28^ use of anti-diabetic medication,^8^ and blood tests of fasting or post-load glucose, haemoglobin HbA_1c_, or other biomarkers.^10,12,29,30^ A meta-analysis of 12 studies found an increased risk of gastric cancer among diabetic patients in East Asian populations, but not Western populations.^31^ The lack of association in the latter populations is in line with the results of the present study of a Western population.

Gastric cardia and non-cardia adenocarcinomas have different aetiology.^3^ Yet, to our knowledge, only two previous studies have analysed diabetes in relation to the risk of gastric adenocarcinoma of these sites separately. A study from the United States showed an increased risk of gastric cardia adenocarcinoma associated with self-reported diabetes, while a study from South Korea found no associations between diabetes and the risk of gastric cardia or non-cardia adenocarcinoma.^10,25^ The results of the latter study are in agreement with the findings of present study.

Lifestyle factors, including smoking, drinking, physical activity and diet, can influence the risk of developing gastric adenocarcinoma.^32^ In the present study, six most relevant confounders were adjusted in the multivariable analyses and further fully adjustment for all the twelve covariates did not change the results much (data not shown). In reality, diabetes patients may change lifestyle habits in response to their diagnosis. We analysed the lifestyle change in a subset of participants (*n* = 1062) who declared no diabetes at the baseline but developed diabetes during follow-up. These participants tended to adopt a healthier diet, i.e., increase the intake of fresh fruit and vegetables and decrease the intake of salt and total energy, and quit smoking after their diagnosis of diabetes (Supplementary Table 1). These lifestyle changes might protect these patients from developing gastric adenocarcinoma and thus counteract or conceal a potential association between diabetes and gastric adenocarcinoma.^33,34^

In summary, this Swedish cohort study, with detailed exposure information, adjustment for several potential confounders and long and complete follow-up, indicates no association between prediabetes or diabetes and risk of gastric adenocarcinoma.

## Supplementary information

Supplementary table 1

## Data Availability

The questionnaires used for VIP and MONICA could be achieved online (VIP https://www.umu.se/enheten-for-biobanksforskning/forskning/northern-sweden-health-and-disease-study/; and MONICA https://www.umu.se/forskning/projekt/monica-projektet/monica-undersokningen-i-norra-sverige2/ accessed Dec. 9th, 2018). Access to the datasets analysed in the current study could be realised by application to Umeå University (https://www.umu.se/enheten-for-biobanksforskning/ accessed 2019–01–18). Computing codes was recorded by the first author (JZ) and could be achieved through communication to the corresponding author (SX).
