# Supplementary material for: Prediabetes and diabetes in relation to risk of gastric adenocarcinoma
Source: Br J Cancer. 2019 May 7;120(12):1147–52. doi: 10.1038/s41416-019-0470-1 (PMC6738058; doi:10.1038/s41416-019-0470-1)
Supplement: Supplementary file 1 — Supplementary table 1 [file 41416_2019_470_MOESM1_ESM.docx]

| ***Supplementary Table 1. Lifestyle changes between baseline and after the diagnosis of diabetes among diabetic participants who were diagnosed during follow-up (N = 1 062)*** | | | |
| --- | --- | --- | --- |
| **Lifestyle factor** | Baseline ^a^ | After diagnosis of diabetes ^a^ | *p* value |
| **Body mass index (BMI)** | | | |
| Mean ± standard deviation | 29.8 ± 0.15 | 30.3 ± 0.16 | <0.001 |
| **Daily alcohol consumption, milligrams** | | | |
| Mean ± standard deviation | 102.8 ± 3.9 | 124.8 ± 5.2 | <0.001 |
| **Daily intake of fruit and vegetables, grams** | | | |
| Mean ± standard deviation | 2.55 ± 0.06 | 3.58 ± 0.08 | <0.001 |
| **Daily sodium intake, milligrams** | | | |
| Mean ± standard deviation | 2300 ± 34.0 | 2079 ± 37.1 | <0.001 |
| **Daily total energy intake, kilocalories** | | | |
| Mean ± standard deviation | 1863 ± 23.7 | 1667 ± 24.5 | <0.001 |
| **Smoking, number (%)** | | | |
| Current smoker | 249 (23.7) | 145 (13.9) | <0.001 |
| Ex-smoker | 348 (33.1) | 419 (40.1) |  |
| Non-smoker | 453 (43.1) | 480 (45.9) |  |
| **Physical activity, number (%)** | | | |
| Inactive | 549 (55.1) | 495 (51.9) | 0.159 |
| Active | 447 (44.9) | 458 (48.1) |  |
| ^a^ Lifestyle factors were compared between baseline (entry of the cohort and free of diabetes) and later visits when these participants reported that they had physician-diagnosed diabetes. The data were collected from the questionnaires completed at these two time points. The means of the continuous variables BMI, daily alcohol consumption, daily intake of fruit and vegetables, daily intake of sodium and daily intake of total energy were compared by paired t-test, while proportions of the tobacco smoking habits and physical activities were compared by chi-squared test. P-values <0.05 were considered statistically significant. | | | |
